# Comparison of two serum free light chain assays for the diagnosis of primary plasma cell malignant proliferative disease

**DOI:** 10.1002/hsr2.113

**Published:** 2019-02-05

**Authors:** Yang Yang, Xiaoyan Han, Gaofeng Zheng, Zhen Cai

**Affiliations:** ^1^ Bone Marrow Transplantation Center, The First Affiliated Hospital Zhejiang University School of Medicine Hangzhou China

**Keywords:** free light chains, immunofixation electrophoresis, method comparison, monoclonal plasma proliferative disorders, sensitivity

## Abstract

**Background and aims:**

The serum free light chain assay (sFLC) is well established for aiding in the diagnosis, prognosis, and monitoring of plasma cell proliferative disorders. There are currently two commercially available sFLC immunoassays, Freelite, based on polyclonal antibody technology, and N Latex FLC, based on monoclonal antibodies. This study aimed to compare the analytical and clinical performance of these two assays in a Chinese population.

**Methods:**

This study included 74 consecutive patients who were newly diagnosed with monoclonal gammopathies (MGs) including multiple myeloma (MM), AL amyloidosis, and light chain deposition disease (LCDD) between January 2014 and May 2015 at the First Affiliated Hospital of Zhejiang University. Alongside serum and urine electrophoresis analysis, the serum samples were retrospectively tested with both sFLC assays according to the manufacturers' instructions.

**Results:**

The two sFLC assays showed a moderate correlation for κFLC (Passing‐Bablok slope = 0.645, coefficient of determination (*R* ^2^) = 0.83, and Spearman coefficient = 0.904). However, for λFLC, a poor correlation was found (Passing‐Bablok slope = 0.690, *R* ^2^ = 0.39, and Spearman coefficient = 0.852). The concordance rate of κFLC, λFLC, and κ/λ FLC ratio were 83.78%, 75.68%, and 86.49%, respectively. The clinical sensitivity of the κ/λ ratios were 83.8% for the Freelite assay and 75.7% for the N Latex FLC assay.

**Conclusion:**

Although the concordance and the clinical sensitivity of the two assays appeared comparable, a number of discrepancies were observed. There is a low correlation between the two assays in clinical practice, suggesting that the assays are not equivalent and, thus, current IMWG guidelines, based on Freelite, cannot be cross‐applied to N Latex FLC.

## 1 INTRODUCTION

Monoclonal plasma proliferative disorders include monoclonal gammopathy of undetermined significance (MGUS), solitary plasmacytoma, multiple myeloma (MM), and AL amyloidosis (AL).^1^ In the past, tests for measuring the circulating monoclonal immunoglobulins, such as serum electrophoresis and immunofixation, have been used alongside urine electrophoresis for the identification of such disorders.^1^, ^2^, ^3^ However, these traditional methods are not sensitive enough to identify nonsecretory MM, many AL patients, and other light chain disorders.^1^, ^3^, ^4^, ^5^

In 2001, a new assay based on the use of polyclonal antisera for the detection of serum free light chains (sFLCs) was developed (Freelite; The Binding Site Group Ltd, UK).^6^ The Freelite assay can accurately detect and quantify both kappa (κ) and lambda (λ) free light chains (FLC) through polyclonal antibodies recognizing a variety of FLC epitopes. The ratio of κ/λ FLC is a sensitive marker of monoclonality, which is key to the clinical utility of the assay. Because of the greater analytical sensitivity of the Freelite assay for identifying monoclonal sFLC, the International Myeloma Working Group (IMWG) have recommended that sFLC testing is included as part of the screening algorithm for MM and related disorders, alongside serum protein electrophoresis (SPE) and serum immunofixation electrophoresis (IFE).^1^, ^7^ The IMWG recently updated the MM diagnostic criteria to include biomarkers of malignancy (also known as the SLiM criteria), which include an involved/uninvolved Freelite serum FLC ratio greater than or equal to 100 (involved FLC should more than 100 mg/L).^7^ This update means that asymptomatic patients, without evidence of related end organ damage (CRAB criteria), can be diagnosed with MM and start therapy if they have one of the SLiM criteria, alongside 10% bone marrow plasma cells or plasmacytoma.

Recently, another sFLC test, based on monoclonal antibodies, became available (N Latex FLC, Siemens, Germany).^8^ Only a small number of studies have compared the diagnostic utility of the two assays.^9^, ^10^, ^11^ This retrospective study is the first such study performed in China, and it aimed to compare the performance of the Freelite and N Latex FLC assays for the diagnosis of monoclonal plasma proliferative disorders.

## 2 METHODS

### 2.1 Patient samples

Consecutive patients who were newly diagnosed with symptomatic monoclonal gammopathies (MGs) including MM, AL amyloidosis, and light chain deposition disease (LCDD) between January 2014 and May 2015 at the First Affiliated Hospital of Zhejiang University (China) were recruited for this study. Repeat samples were not included in the study, and only one sample was permitted per patient. Only the remnant serum samples after routine testing were analyzed. Seventy‐four remnant serum specimens were stored at −70°C after routine testing, so that the FLC test could be performed retrospectively. At the time of the FLC analysis, the samples were thawed once and thoroughly mixed prior to analysis. This study was approved by the First Affiliated Hospital of Zhejiang University (China) Human Research Ethics Committee. Written informed consent was obtained from all participating patients.

### 2.2 Immunofixation electrophoresis

Serum and urine IFE analyses were performed using the Helena SPIFE 3000 system (Helena, USA), according to the manufacturer's instructions. All results were evaluated by two independent readers.

### 2.3 FLC assays

Two FLC assays for FLC κ and λ in serum were evaluated: Freelite assays (The Binding Site Group Ltd., UK; catalog number: LK016.IM/LK018.IM, lot number: κ344785/λ349269), using a polyclonal antibody‐based method, and N Latex FLC assays (Siemens Healthcare Diagnostics GmbH, Germany; catalog numbers OPJA03/OPJB03, lot numbers: κ473123/λ473223), using a monoclonal antibody‐based method. Freelite assays were performed on a Beckman Coulter Immage800 (Beckman Coulter, Inc. USA). Freelite assays were performed at both the initial dilution and at 1/250 dilution to preclude the possibility of false negatives caused by antigen excess (as per the manufacturer's instructions). N Latex FLC assays were performed on Siemens BNII (Siemens Healthcare Diagnostics GmbH, Germany). The N Latex FLC assay was subjected to a prereaction step for 2 minutes prior to the assay, during which samples were diluted in the presence of a high concentration of antigens, thereby reducing the risk of false negatives caused by antigen excess. Both assays were performed according to the manufacturers' instructions. Serum dilutions, both initial and subsequent (where results were outside of the reportable range), were performed as recommended by the manufacturers. Supplied commercial controls were included on each run of the Freelite assays and N Latex FLC assays.

sFLC concentrations and ratios were considered abnormal if they were outside the reference ranges provided by the manufacturers, which were as follows: Freelite κ, 3.3 to 19.4 mg/L; Freelite λ, 5.7 to 26.3 mg/L; Freelite κ/λ ratio, 0.26 to 1.65; N Latex FLC κ, 6.7 to 22.4 mg/L; N Latex FLC λ, 8.3 to 27 mg/L; N Latex FLC κ/λ ratio, 0.31 to 1.56.

### 2.4 Statistics

The Clinical and Laboratory Standards Institute (CLSI)(EP09‐A2‐IR) guide to Method Comparison and Bias Estimation using Patient Samples (EP‐09‐A2‐IR) states that an *R* ^2^ ≥ 0.95 is required for establishing that two assays are equivalent.^12^ We, therefore, performed Passing‐Bablok regression and Spearman correlation analysis to determine this parameter, and bias was determined by the Bland‐Altman method. Statistical analyses were performed using the Statistical Package for the Social Sciences software version 16 (SPSS Inc., Chicago, IL, USA). Agreement between the two assays, relative to their respective reference ranges, was assessed by Cohen κ statistic, and concordance analysis was performed in Microsoft Excel (Microsoft Office professional Plus 2010).

## 3 RESULTS

### 3.1 Patient characteristics

Samples were from 74 patients, who were recently diagnosed with an MG using commonly accepted criteria.^1^, ^13^ The median age was 63 years (interquartile range 56.5 to 68). The male to female ratio was 1:1. Clinical diagnoses of the patients, determined by local physicians, were as follows: 70 were diagnosed with MM, three with AL, and one patient with LCDD. Of the samples, 90.5% (67/74) had detectable paraprotein confirmed by serum or urine IFE, six samples were negative and were diagnosed as nonsecretory MM (NSMM), and one sample from an AL patient was negative. Of the remaining MM patients, 14 were classified as light chain MM (LCMM) and 50 as intact immunoglobulin multiple myeloma (IIMM).

### 3.2 Correlation between the two FLC assays

**Figure 1:**
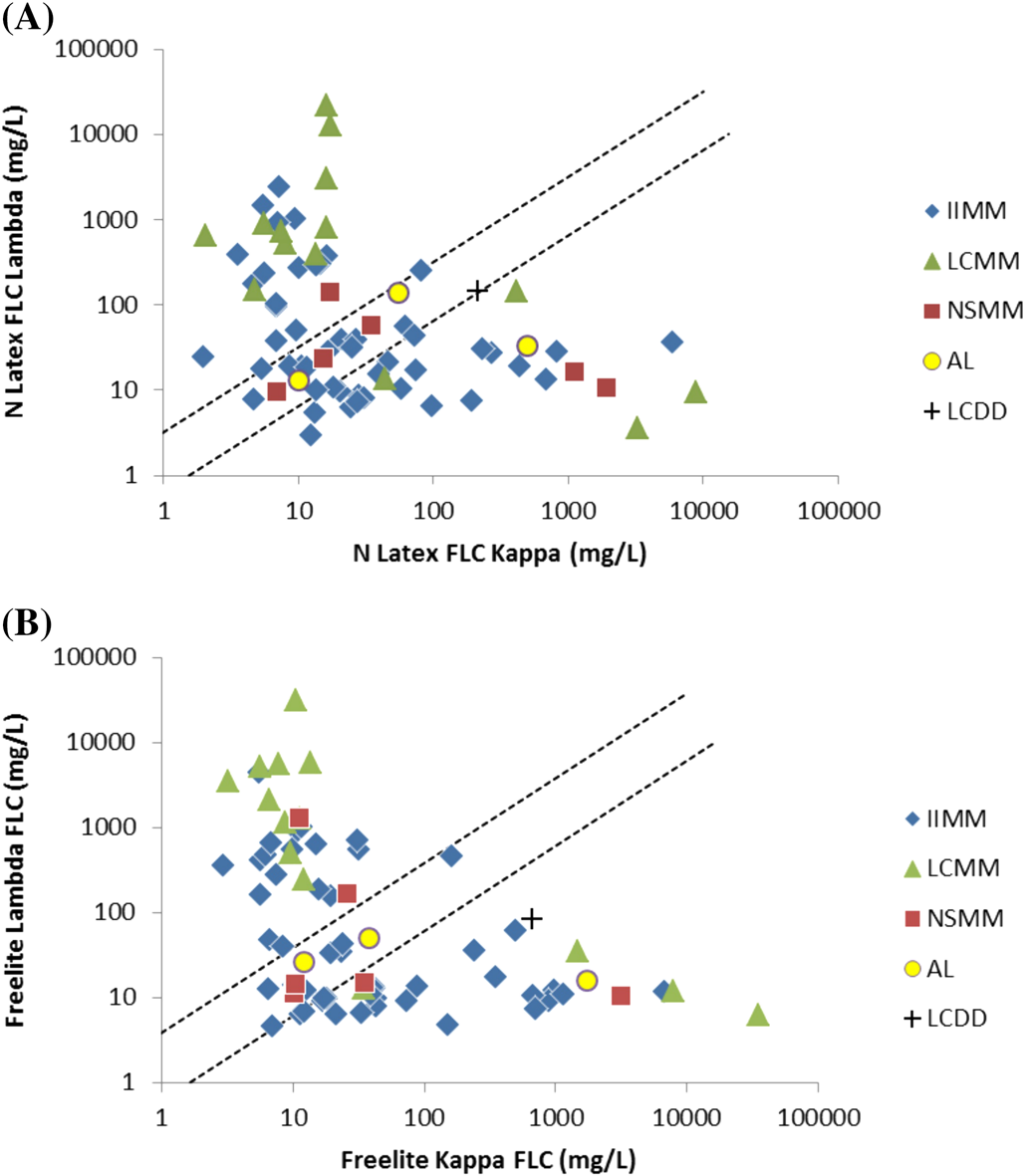
Kappa and lambda distribution scatter diagram for A, N Latex FLC assays and B, Freelite assays. Dotted lines indicate reference ranges

The recommended reference ranges for the Freelite assay and the N Latex FLC assay were used for the analysis. The concentrations of the κ and λ sFLCs as measured by the two assays are plotted in Figure 1.

The range of κ FLC values, as measured by the Freelite assay, was 2.94 to 35 250.0 mg/L and was, in general, higher than those obtained in the N Latex FLC assay (2.0 to 8930.0 mg/L). Likewise, the values for λ FLC measured by the Freelite assay (4.6 to 31 000 mg/L) were also higher than those in the N Latex FLC assay (3.04 to 21 800 mg/L).

**Figure 2:**
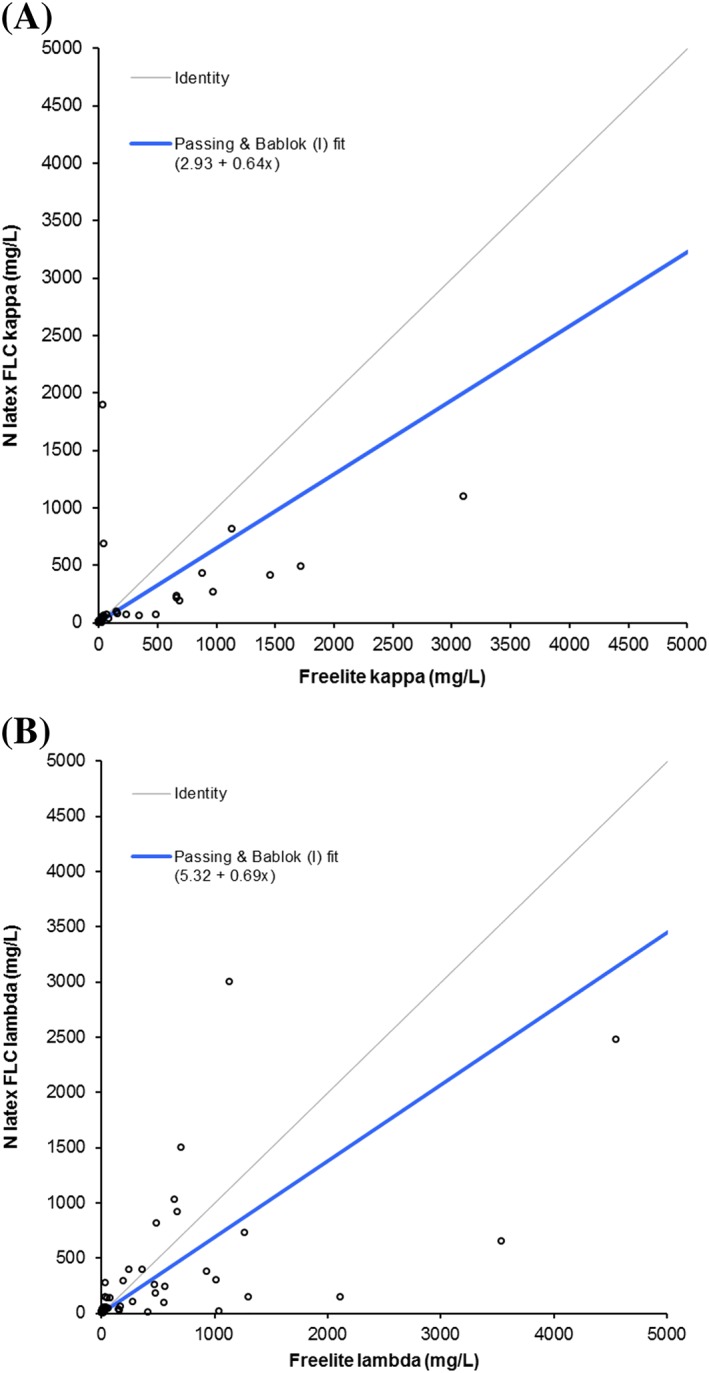
Passing‐Bablok regression analysis comparing A, FLC kappa and B, lambda measurements by Freelite and N Latex FLC. Passing‐Bablok analysis was performed on all 74 samples. However, to optimize axes scaling and presentation of the results, data points for three kappa FLC samples (patient 27: [35 250 mg/L, 8930 mg/L]; patient 90: [7800 mg/L, 3290 mg/L]; and patient 65: [6750 mg/L, 5880 mg/L]—[Freelite, N Latex FLC] respectively) and two lambda FLC samples (patient 8: [31000 mg/L, 12900 mg/L] and patient 108: [5700 mg/L, 21800 mg/L]—[Freelite, N Latex FLC] respectively) are not shown, but are included in Figure S1

**Figure 3:**
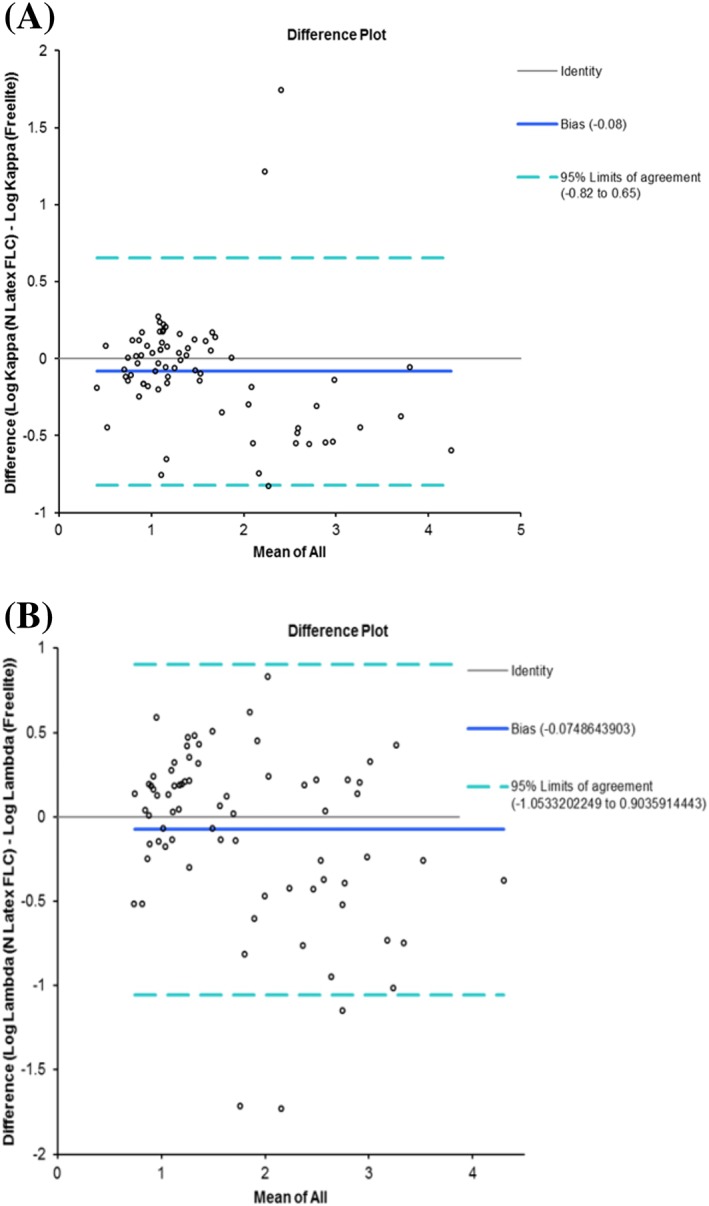
Bland‐Altman plots for A, κFLCs and B, λFLCs, respectively. The analysis was performed using log transformed data

The method comparison between the two assays (Figure 2) showed a moderate correlation for κ FLC values; the slope for the Passing‐Bablok regression analysis was 0.645, and the coefficient of determination (*R* ^2^) was 0.83, with a Spearman coefficient of 0.904. However, for λ FLC values, the Passing‐Bablok regression analysis determined the slope to be 0.690, with an *R* ^2^ of 0.39 and a Spearman coefficient of 0.852, indicating a poor correlation. Bland‐Altman analysis showed a bias of −0.08 for κFLC (95% limits of agreement, −0.82 to 0.65) and −0.075 for λFLC (95% limits of agreement, −1.05 to 0.95) (Figure 3), although it is interesting to note that for both κ and λ FLC measurements at higher concentrations, there seems to be a more pronounced underreading by N Latex FLC compared with Freelite.

### 3.3 Concordance between the two FLC assays

The data was analyzed according to the manufacturer's reference ranges and categorized into low (below the reference range), normal (within the reference range), and high (above the reference range), to assess qualitative concordance.

**Figure 4:**
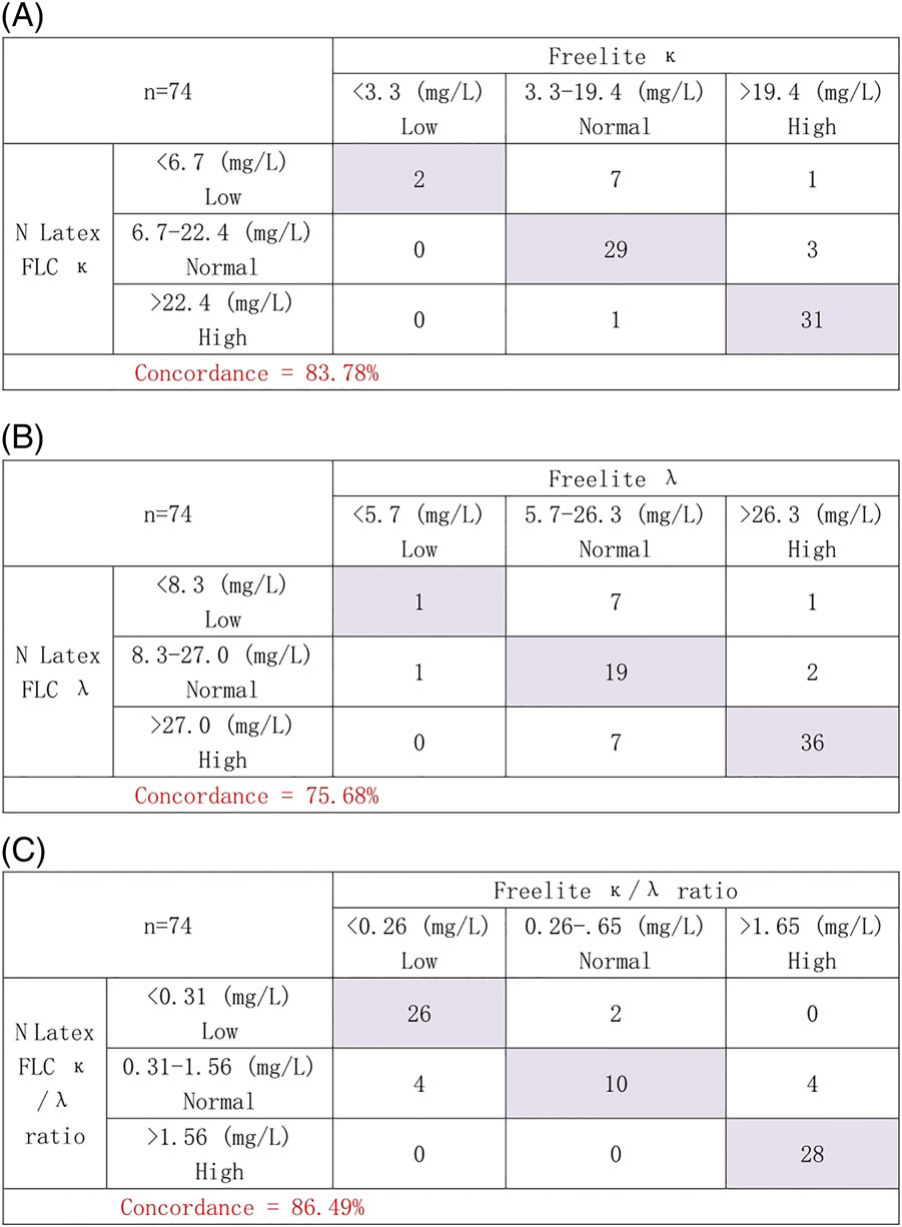
Concordance analysis of A, FLC κ, B, FLC, and C, κ/λ FLC ratio of the N Latex FLC and the Freelite assays

The concordance rate of κ FLC, λ FLC, and κ/λ FLC ratio were 83.78%, 75.68%, and 86.49%, respectively (Figure 4). Concordance for κ FLC was good, with Cohen kappa of 0.75, but it was only moderate for λ FLC, with a Cohen kappa of 0.644. For κ/λ FLC ratio, the Cohen kappa was 0.59, based on whether the results were simply normal (within the manufacturer's reference range for the κ/λ FLC ratio) or abnormal (outside the reference ranges) and, thus, highlighting only moderate concordance between the two assays.

For 10 patients, the results for the κ/λ FLC ratio were discordant (13.5% of patients) (Table 1). Eight had an abnormal Freelite κ/λ FLC ratio and a normal N Latex FLC ratio, whereas two were normal according to Freelite and abnormal according to N Latex FLC. Two of the patients with discordant results had negative electrophoresis, and in both cases, the monoclonal FLCs were identified by the Freelite assay, but the N Latex FLC ratio was within the normal range. The Freelite results were in agreement with the clinical diagnosis of NSMM and LCDD for these patients.

**Table 1:** Samples with discordant serum free light chain (sFLC) ratios

Sample No	Clinical Diagnosis	sIFE	N latex FLC			Freelite		
			κFLC, mg/L	λFLC, mg/L	Ratio	κFLC, mg/L	λFLC, mg/L	Ratio
1	IIMM	IgG/K	62.5	56.4	1.11	350	17.6	19.89
6	IIMM	IgG/K	13.6	10.2	1.33	12.0	7.1	1.70
11	IIMM	IgA/L	6.9	38.3	0.18	5.7	14.3	0.40
26	NSMM	Negative	34.2	58.2	0.59	25.7	172	0.15
35	IIMM	IgG/K	67.9	49	1.39	241	37	6.51
43	IIMM	IgG/L	21	39.4	0.53	19.3	158	0.12
47	LCDD	Negative	218	142	1.54	665	82.1	8.10
58	IIMM	IgG/L	10.7	19.5	0.55	9.9	1045	0.01
62	IIMM	IgA/L	4.7	8.04	0.59	5.6	417.5	0.01
74	IIMM	IgA/L	5.3	17.8	0.30	6.9	4.6	1.49

Abbreviations: FLC, free light chains; IIMM, intact immunoglobulin multiple myeloma; LCDD, light chain deposition disease; NSMM, nonsecretory multiple myeloma; sIFE, serum immunofixation electrophoresis.

### 3.4 Clinical sensitivity of the κ/λ ratio of FLC assays

On the basis of the clinical diagnosis, the sensitivity of the κ/λ FLC ratio of the two methods to detect the presence of monoclonal FLCs was calculated. For the Freelite assay, sensitivity was 83.8% (62/74), and for N Latex FLC assay, it was 75.7% (57/74), indicating the Freelite assay has a higher clinical sensitivity. One patient with renal impairment (creatinine greater than 177μmol/L) had a normal κ/λ FLC ratio by both assays because of an elevation in both the κ and the λ FLC. However, by applying the Freelite renal reference range (0.37 to 3.1),^14^ this patient would now be classified as having an abnormal ratio by Freelite (0.34). This result is in line with the clinical assessment and electrophoresis results. There is no equivalent renal reference range validated for the N Latex FLC assay, so this patient's monoclonal FLCs would not be identified by the N Latex FLC assay.

### 3.5 Proportion of patients with measurable FLC

By current IMWG consensus guidelines, one of the criteria for measurable disease in MM is defined as an involved free light chain (iFLC) greater than 100 mg/L.^3^ In this study, we identified more MM patients with measurable disease by the Freelite assay (61.97%) than with the N Latex FLC assay (52.11%). There were five oligosecretory MM patients in this study; all five had measurable disease by Freelite; however, one of these patients did not have measurable disease measured by N Latex FLC.

## 4 DISCUSSION

A number of studies have shown that sFLC analysis plays a valuable role in the diagnosis, prognosis, and response assessment of plasma cell dyscrasias.^1^, ^3^, ^15^, ^16^, ^17^ Such studies have resulted in the inclusion of sFLC analysis in the IMWG guidelines.^1^, ^3^, ^16^ These studies are based on sFLC data measured by the polyclonal Freelite assay. With the introduction of a second method for measuring sFLCs, based on monoclonal antibodies, it is important to determine whether this method is equivalent to the polyclonal FLC immunoassay. In order to assess these methods, in this study, we compared the two FLC assays using samples from 74 patients diagnosed with MM, AL, or LCDD.

Correlation analysis showed that the two assays are not equivalent, with modest agreement. These results are consistent with a number of other studies.^9^, ^10^, ^18^ According to CLSI Guide to Method Comparison (EP09‐A2‐IR), the square of linear regression coefficient (*R* ^2^) should be greater than or equal to 0.95 for two methods to be considered equivalent. In our study, the *R* ^2^ values are all lower than 0.95, especially the λ FLC value comparison, meaning that Freelite and N Latex FLC cannot be considered equivalent under these criteria.

The Freelite assay has shown greater sensitivity than the N latex FLC assay in the detection of monoclonal FLCs in serum.^9^ We observed that in samples with extremely high levels of FLC, the difference between the results reported by the two assays was more pronounced. de Kat Angelino et al^19^ speculated that overestimation of FLC by Freelite may be due to polymerization of monoclonal FLCs. On the other hand, polymerization of FLCs could hide epitopes, leading to an underestimation of FLC by the N Latex FLC assay.

We found eight samples in which the monoclonal FLCs were not detected by the N Latex FLC assay (three IgGκ, three IgGλ, one κ FLCs, and one λFLCs; six IIMM, one LCDD, and one NSMM) and two by Freelite (two IgAλ; IIMM). Unlike other studies comparing the two FLC assays, we observed no difference in the FLC isotype of the patients missed.^9^, ^10^, ^18^

Of the five NSMM patients included in this study, there were two patients in whom neither assay was able to detect the presence of monoclonal sFLCs, possibly indicating that these patients were true nonsecretors. However, in the three remaining NSMM patients, Freelite identified the presence of monoclonal FLCs in all three patients, whereas N Latex FLC was unable to identify the monoclonal FLCs in one of them. This inability to identify the monoclonal FLCs could result in a delayed diagnosis and difficulty in monitoring the patient's response to treatment. Together, these data suggest that the polyclonal antibody reagent has clinical sensitivity that is superior to the monoclonal antibody reagent. We speculate that because the Freelite assays are based on sheep polyclonal antisera, these assays should recognize the majority of polymorphic monoclonal FLCs. On the other hand, the N Latex FLC is based on monoclonal antibodies, and because of the limited epitope specificity, is unlikely to recognize all monoclonal FLC clones.

One of the five patients with renal damage had an abnormal κ/λ FLC ratio if the Freelite renal reference range (0.37 to 3.1) was utilized.^14^ The clinical symptoms and other findings led to a diagnosis of MM, confirming the Freelite results. Jacobs et al^20^ studied patients with renal impairment due to chronic kidney disease and demonstrated the N Latex FLC assay does not need distinct reference ranges in patients with renal failure. We agree that the renal reference range could improve the sensitivity and specificity of the Freelite FLC assay; such a ratio is yet to be determined for N Latex FLC assay.

The measurable disease criteria are based on studies using the Freelite assays.^3^ Our data suggests such criteria may not be applicable to N Latex FLC, as fewer patients had measurable disease as determined by N Latex FLC. There were five oligosecretory in 64 MM patients (except for NSMM), on the basis of electrophoresis results. It is interesting that one MM patient had more than 100 mg/L FLC by Freelite but lower than 100 mg/L by N Latex FLC. So, in considering measurable disease, Freelite appears to have more sensitivity. The data presented here indicates that because of the differences between the involved FLC concentrations measured by the two assays, guidelines either need to consider different cutoffs for the different FLC assays or a single cutoff for both assays that includes as many patients with measurable disease as possible.

Some limitations in the present study should be noted. First, our sample size was relatively small, although our conclusions are in accordance with other studies. Secondly, this is a retrospective study, and the two FLC assays were performed on frozen serum and run on different platforms. These factors may introduce bias into the study, but we believe they should not significantly affect the comparison of the two FLC assays.

Our clinical study demonstrated that although the two assays perform similarly in the detection of FLC, a number of discrepancies were observed. There is a low correlation between the two assays in clinical practice, suggesting the assays are not equivalent. Therefore, the current international guidelines based on the Freelite assays cannot be cross‐applied to the N Latex FLC assay. More data are required to establish FLC criteria for N Latex FLC assay.

## FUNDING

This work is supported by the Natural Science Foundation of Zhejiang Province (LY18H080001). The funder played no role in study design; collection, analysis, and interpretation of data; writing of the report; or the decision to submit the report for publication.

## CONFLICTS OF INTEREST

The authors declare no conflicts of interest relevant to this study.

## AUTHOR CONTRIBUTIONS

Conceptualization: Yang Yang, Zhen Cai

Formal analysis: Xiaoyan Han

Resources: Gaofeng Zheng

Validation: Xiaoyan Han

Writing—original draft: Yang Yang

Writing—review and editing: Zhen Cai, Yang Yang

Dr Zhen Cai had full access to all of the data in this study and takes complete responsibility for the integrity of the data and the accuracy of the data analysis.

## Supporting information


**Figure S1.** Passing Bablok regression analysis comparing (A) FLC kappa (A) and (B) lambda measurements by Freelite and N Latex FLC. Passing Bablok analysis was performed on all samples.
