# Supplementary figures and images for: Comparison of two serum free light chain assays for the diagnosis of primary plasma cell malignant proliferative disease
Source: Health Sci Rep. 2019 Feb 5;2(4):e113. doi: 10.1002/hsr2.113 (PMC6482328; doi:10.1002/hsr2.113)

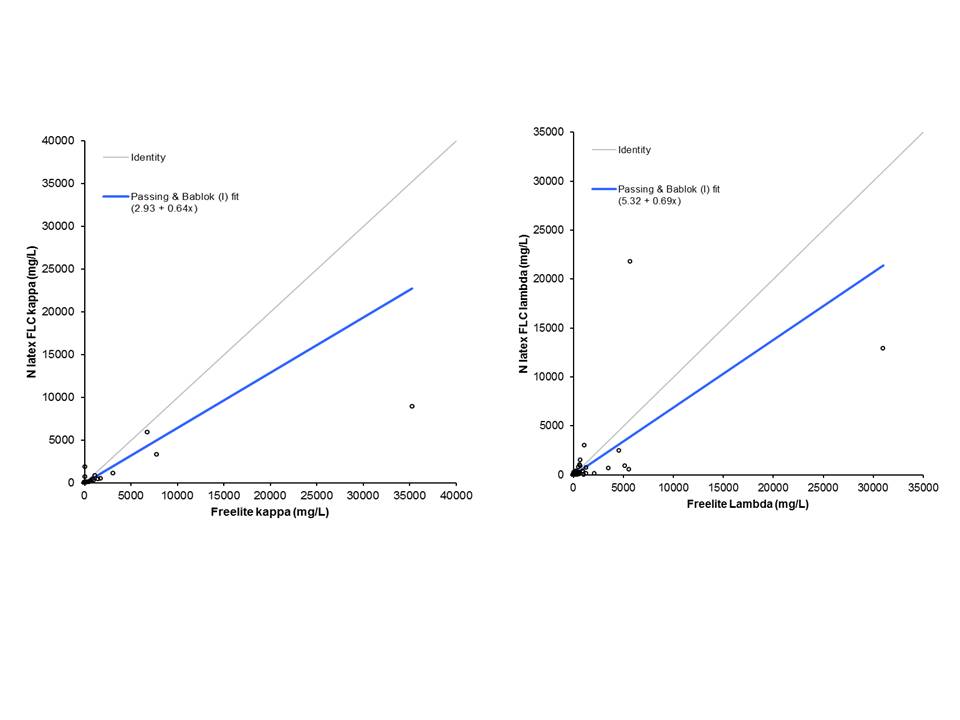

Supplement: Supplementary file 1 — Figure S1. Passing Bablok regression analysis comparing (A) FLC kappa (A) and (B) lambda measurements by Freelite and N Latex FLC. Passing Bablok analysis was performed on all samples. [file HSR2-2-e113-s001.jpg]
